# Conformational exchange in the potassium channel blocker ShK

**DOI:** 10.1038/s41598-019-55806-3

**Published:** 2019-12-17

**Authors:** Naoto Iwakawa, Nicola J. Baxter, Dorothy C. C. Wai, Nicholas J. Fowler, Rodrigo A. V. Morales, Kenji Sugase, Raymond S. Norton, Mike P. Williamson

**Affiliations:** 1 Department of Molecular Engineering, Graduate School of Engineering, Kyoto University, Kyoto-Daigaku Katsura, Nishikyo-Ku, Kyoto 615-8510 Japan; 2 Department of Molecular Biology and Biotechnology, University of Sheffield, Western Bank, Sheffield, S10 2TN UK; 3 Medicinal Chemistry, Monash Institute of Pharmaceutical Sciences, Monash University, Parkville, Victoria 3052 Australia; 4 Present Address: CSL Limited (Bio21), 30 Flemington Road, Parkville, Victoria 3010 Australia; 5 ARC Centre for Fragment-Based Design, Monash University, Parkville, Victoria 3052 Australia

**Keywords:** Peptides, NMR spectroscopy

## Abstract

ShK is a 35-residue disulfide-linked polypeptide produced by the sea anemone *Stichodactyla helianthus*, which blocks the potassium channels Kv1.1 and Kv1.3 with pM affinity. An analogue of ShK has been developed that blocks Kv1.3 > 100 times more potently than Kv1.1, and has completed Phase 1b clinical trials for the treatment of autoimmune diseases such as psoriasis and rheumatoid arthritis. Previous studies have indicated that ShK undergoes a conformational exchange that is critical to its function, but this has proved difficult to characterise. Here, we have used high hydrostatic pressure as a tool to increase the population of the alternative state, which is likely to resemble the active form that binds to the Kv1.3 channel. By following changes in chemical shift with pressure, we have derived the chemical shift values of the low- and high-pressure states, and thus characterised the locations of structural changes. The main difference is in the conformation of the Cys17-Cys32 disulfide, which is likely to affect the positions of the critical Lys22-Tyr23 pair by twisting the 21–24 helix and increasing the solvent exposure of the Lys22 sidechain, as indicated by molecular dynamics simulations.

## Introduction

ShK is a polypeptide toxin produced by the sea anemone *Stichodactyla helianthus*^1^. It contains 35 residues and six cystines, linked together in three disulfide bonds^2^. ShK is a potent inhibitor of voltage-gated potassium channels, in particular Kv1.1 and Kv1.3, which it blocks with IC_50_ values of 16 and 10 pM, respectively^3^. This led to interest in developing it as a therapeutic agent, particularly one with greater selectivity for the Kv1.3 channel, which is a validated target in autoimmune disease^4–7^, over Kv1.1 and Kv1.2, which have roles in the central nervous system. The result of this sustained effort was the development of an analogue of ShK (ShK-186, or dalatazide), which is amidated at the *C-*terminus and has a phosphotyrosine at the *N*-terminus^3^. This analogue is 100-fold selective for Kv1.3 over all other targets. ShK-186 has been shown to be effective against a range of autoimmune diseases in animal and human studies^8,9^, has completed Phase 1b clinical trials, and is being considered as a potential treatment for diseases such as psoriasis and multiple sclerosis.

Considerable effort has gone into identifying structural elements in ShK that are critical for potassium channel blockade. Alanine scanning mutagenesis suggested that the key residues for binding to the Kv1.2 channel are Lys22 and Tyr23^10,11^, with Lys22 being the most important for Kv1.3^12^. Comparison of NMR^13^ and X-ray^14^ structures of ShK reveals small but possibly significant structural differences^14^. In both structures, the important Tyr23 sidechain is partially buried within the structure, and has only 23% solvent-accessible surface area^15^. There is no crystal structure of Kv1.3, but there is a structure of the bacterial potassium channel KcsA, which has been used previously in computational studies as a model for Kv1.3^12,16^. Docking of this Kv1.3 model with ShK and with ShK-Dap22 (in which Lys22 is replaced by diaminopropionic acid) suggested that Lys22 binds to a negatively-charged region in the pore^17^. A detailed analysis of binding energies in this study led to the hypothesis that there may be a conformational change when ShK binds to the Kv1.3 channel, supporting a suggestion made earlier on the basis of NMR linewidths^13^. A subsequent modelling study also concluded that a conformational change may be required for ShK binding^18^, although we should note that another modelling study in 2011 concluded that a conformational change on binding was not required^19^.

To explore the possibility of conformational changes associated with channel binding, NMR studies of conformational exchange were undertaken. A set of relaxation dispersion measurements indicated conclusively that conformational change is occurring^15^, involving a minor conformation that is probably less structured than ShK and has increased solvent exposure of Tyr23. The lifetime of this alternative state was in the sub-millisecond range. It was not possible to characterise the exact motion responsible, although it was speculated that the motion could be induced by changes in the disulfide bridges. Subsequently a more sophisticated relaxation analysis was carried out^20^, which concluded that the disulfide network could be involved in the conformational exchange, but was unable to more specifically identify the mechanism. There is an interesting contrast between ShK and HsTX1, which is over 2000-fold more selective for binding to Kv1.3 over Kv1.1, has one extra disulfide bond, and is much more rigid than ShK^21^.

In this work, we have characterised the alternative state of ShK using high-pressure NMR. High hydrostatic pressure favors conformations that have smaller partial molar volume^22^. These tend to be partially unfolded forms, and every high-pressure conformer studied so far has proven to be functionally important^23^. It is possible to carry out conventional structure determinations on proteins at high pressure^24^, but such studies require high populations of the pressure-induced state, which is usually not experimentally accessible. We recently developed an alternative approach: chemical shifts are followed during a pressure titration, and the changes in chemical shift caused by pressure perturbation are fitted to an equation that allows us to extract the chemical shift values of the low- and high-pressure states, as well as the global Δ*G* and Δ*V* values for the conformational change^25^. These chemical shift values provide structural information. Using this approach, we show that there is a conformational change with pressure in ShK; that the free energy difference between the two states is 1.9 kJ mol^−1^, implying that binding to the potassium channel is easily able to induce conformational change; and that the conformational change is driven by the Cys17-Cys32 disulfide bond, which induces a reorientation of the 21–24 helix, thereby positioning Lys22 and Tyr23 differently on the surface.

## Results

### Fitting of the pressure-dependent shifts

**Figure 1:**
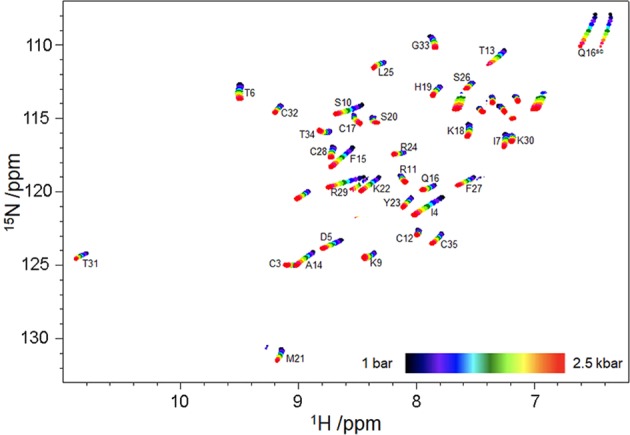
^15^N HSQC spectra of ShK acquired at pressures from 1 to 2500 bar. Unlabeled peaks are from sidechains.

^13^C, ^15^N and ^1^H chemical shifts were obtained for most C, N and H nuclei in ShK using HSQC and 2D HNCO measurements taken at regular pressure intervals between 1 and 2500 bar (2500 bar = 250 MPa) (Fig. 1 and Supplementary Fig. S1). Many signals have approximately linear chemical shift changes with pressure, as is often observed^26^. The shift changes are due to a gradual compression of the protein with pressure, with compressibilities that are almost independent of pressure. A few signals have markedly curved shift changes, which indicate the presence of an alternative state^23,26^. These curved shift changes have most often been fitted to a quadratic expression, *δ* = *a* + *bp* + *cp*^2^, where the pressure is *p* and the curvature coefficient *c* tends to report on conformationally flexible regions, typically close to cavities or buried water molecules^24^. While this is a useful analysis, there is no direct relationship between the coefficients and any physical or structural parameter. We therefore recently introduced a different expression for the measured shift, given by Eq. (1):

1 $$\delta =\frac{({\delta }_{1}^{0}+p\Delta {\delta }_{1})+({\delta }_{2}^{0}+p\Delta {\delta }_{2})\,exp\,(\,-\,[\Delta G+p\Delta V]/RT)}{1+exp\,(\,-\,[\Delta G+p\Delta V]/RT)}$$

This equation assumes fast exchange on the chemical shift timescale between form **1** (ground state) and form **2** (pressure-stabilised alternative state), with the populations of the two states given by a Boltzmann distribution dependent on the pressure. *δ*_1_^0^ and *δ*_2_^0^ are the chemical shifts of forms **1** and **2** at ambient pressure; Δ*δ*_1_ and Δ*δ*_2_ are the linear pressure-dependent changes in chemical shift; *p* is the pressure; Δ*G* is the difference in Gibbs free energy between the two states at ambient pressure; and Δ*V* is the change in partial molar volume between the two states. The primary advantage of this expression is that the fitting yields parameters whose values have a physically interpretable meaning.

To improve the reliability of the fitting, the measured pressure-dependent chemical shift values were processed using singular value decomposition (SVD). This is a widely used statistical data reduction technique, which models the experimental data as being comprised of a sum of terms, weighted by a column matrix of singular values. The rank of the singular value matrix **W** (the number of meaningful singular values) reflects the number of components required to account for the experimental measurements. Here, excellent fits to the experimental data could be obtained by using four singular values (see Supplementary Fig. S2). This fits with our model, which assumes four states of the protein: a ground state at ambient pressure; a ground state at high pressure (namely, the same structure but compressed in some anisotropic manner); a pressure-stabilised alternative state at ambient pressure; and a pressure-stabilised alternative state at high pressure. We therefore set all singular values greater than 4 to zero and recalculated a ‘noise-free’ set of experimental shifts, as described^25^.

These shifts were then fitted to a quadratic function. Those signals that fitted least well to the quadratic (the signals with the largest χ^2^ values from the fitting) were used to fit to Eq. (1), the requirement being that all signals should fit to a common Δ*G* and Δ*V*. Different subsets of signals were used to check for a robust fit, resulting in fitted values of Δ*G* = 1.9 ± 0.4 kJ mol^−1^ and Δ*V* = −1.4 ± 0.3 kJ mol^−1^ kbar^−1^. This free energy difference corresponds to a population of the excited state of 30% at ambient pressure and 65% at 2.5 kbar. Given the very strong affinity of ShK for the potassium channel, it is clear that the binding interaction is easily strong enough to select the alternative conformation in a process of conformational selection, as has been previously proposed^15^. The change in partial molar volume is small, implying that the excited state conformation largely retains a folded conformation.

**Figure 2:**
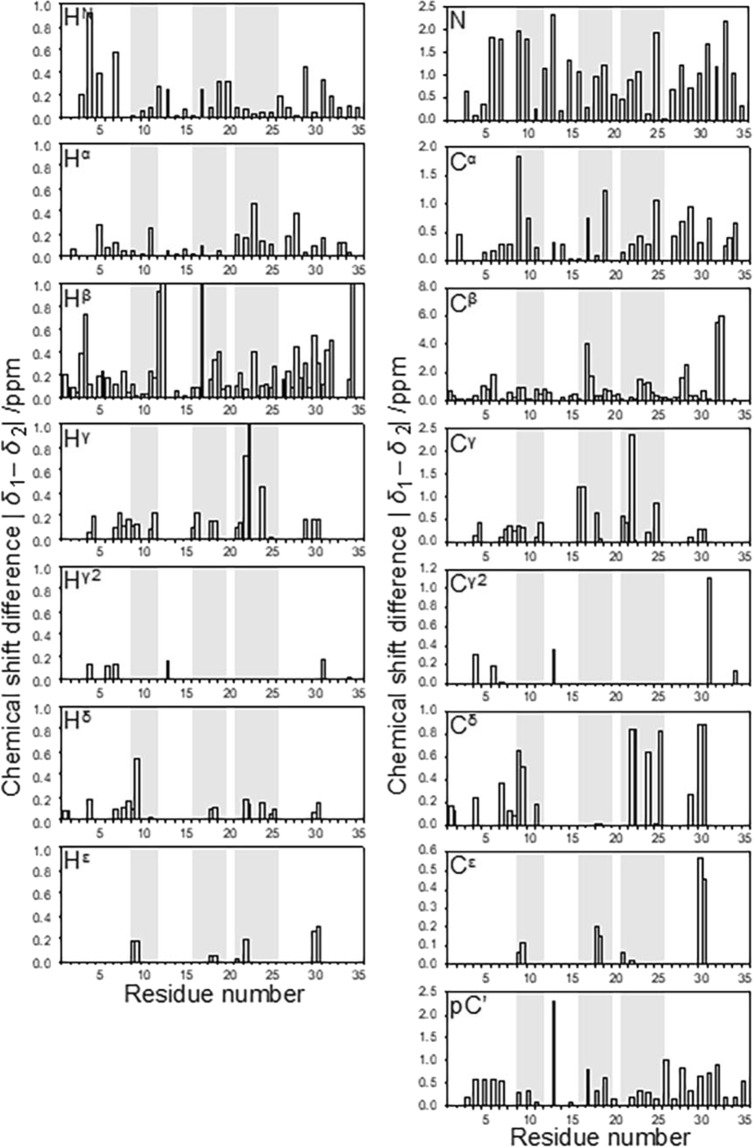
Differences in the fitted values of ^1^H, ^15^N, ^13^C′, ^13^Cα and ^13^Cβ shifts between ground state and pressure-induced excited state. Regions of regular secondary structure are shaded. For carbons that have two protons attached, there are often two different shift values.

Once global Δ*G* and Δ*V* values had been obtained, they were fixed, and signal-specific chemical shift changes were fitted to Eq. (1), to give values of *δ*_1_^0^, *δ*_2_^0^, Δ*δ*_1_ and Δ*δ*_2_ for each nucleus (Fig. 2, Supplementary Fig. S3).

### Interpretation of fitted shifts

**Figure 3:**
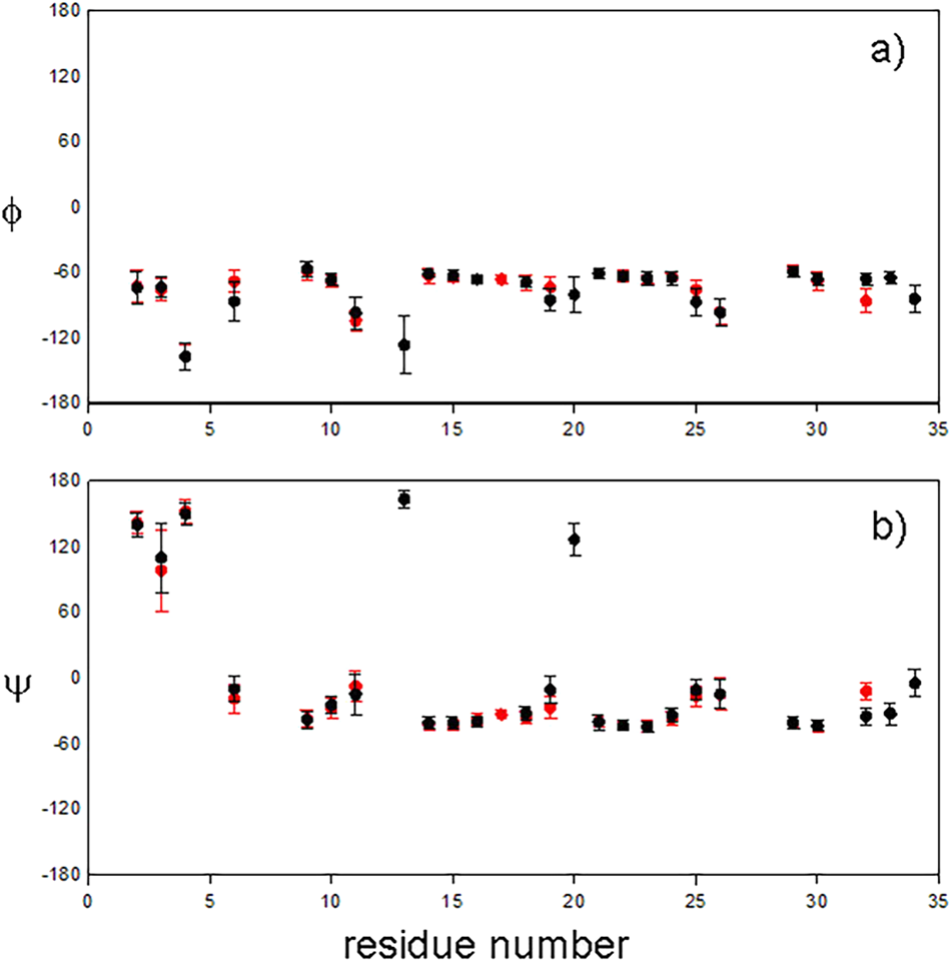
TALOS-N analysis of backbone dihedral angles in ground and pressure-induced excited states of ShK. (**a**) φ, (**b**) ψ backbone dihedral angles. Ground state in black, and excited state in red. Only those residues are shown where the TALOS-N prediction is confident.

Chemical shifts are conformation-dependent, and several methods have been proposed to extract structural information from chemical shifts^27,28^. Here we used the TALOS-N program^29^, which compares backbone chemical shifts to a database and predicts the most likely dihedral angles in the protein. The results of this process are shown in Fig. 3. Despite some fairly large chemical shift changes, most residues are predicted to have backbone dihedral angles that are almost unchanged in the alternative state. The only exceptions, where the predicted change is more than twice the pooled uncertainty, are the φ and ψ dihedral angles for Cys32.

The TALOS-N predictions are based on all backbone shifts (^1^H_N_, ^15^N, ^13^Cα, ^13^Cβ, ^13^C′ and ^1^Hα). Of these, the most strongly conformation-dependent are those of ^13^Cα and ^13^Cβ, which are shown in Fig. 2. The largest changes are seen for residues Cys32 and Cys17, which are linked in a disulfide bridge, strongly implying that the pressure-dependent conformational change involves this disulfide. There is considerable precedent for conformational changes in disulfide bridges, almost all associated with changes in the chirality of the bridge^30^. It is thus likely that the conformational change seen here also involves a change in chirality of the bridge.

TALOS-N can be used to predict χ_1_ sidechain dihedral angles as well as backbone dihedral angles. However, in this case it was unable to make useful predictions for cystine residues. We therefore used DISH^31^, a database and program specifically designed for cyst(e)ines. The program predicts (with a confidence score of approximately 70%) that none of the cystines undergoes a conformational change with pressure, except for Cys32, which is predicted to change χ_1_ from 180° to −60°, and χ_2_ from +60° to −60°, with some change in the backbone dihedral angles also.

**Figure 4:**
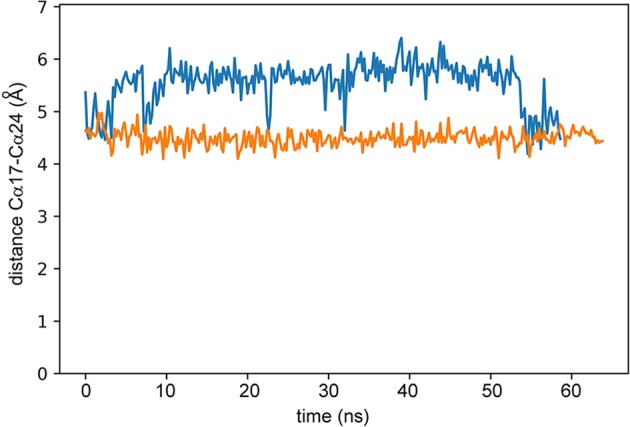
The distance between Cα of Cys17 and Cα of Arg24, in an enhanced sampling molecular dynamics trajectory in which each iteration represents 0.2 ns. The blue line is for a negative Cys17-Cys32 χ_3_, as in the crystal structure, while the orange line is for a positive χ_3_. The downward excursions of the distance (eg at 22.6 ns for the negative angle) are due to changes in the position of the disulfide, rather than a change in the chirality of the angle.

**Figure 5:**
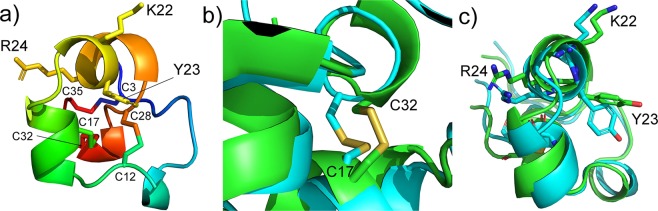
Views of ShK. (**a**) Overall structure of ShK, colored from blue at the *N*-terminus to red at the *C*-terminus, using the PDB file 4LFQ^14^. The six cystines are indicated, plus three residues thought to interact with the Kv1.3 potassium channel (Lys22, Tyr23 and Arg24). (**b**) Closeup view of the C17-C32 disulfide. The structures shown are iterations 200 from the molecular dynamics simulations, with the ground-state (negative χ_3_) in green, and the alternative conformation (positive χ_3_) in cyan. (**c**) End-on view of helix 21–24, showing the positions of Lys22, Tyr23 and Arg24 in iterations 200 from the molecular dynamics simulations. The Cys17-Cys32 disulfide is just visible at the back of the helix at the bottom.

We therefore conclude that there is a conformational change between the ground state and pressure-stabilised state, but that it is small. The most obvious feature is that it involves Cys17 and Cys32, and very likely involves a change in the chirality of the disulfide, accompanied by a change in the orientation of the Cys32 sidechain. We therefore calculated the effect of a change in chirality of the disulfide on the peptide structure, by using molecular dynamics simulations of ShK in explicit solvent, restraining the χ_3_ dihedral angle of the S-S bond to be either −80 ± 30° (as in the crystal structure) or +80 ± 30°. The two trajectories produce similar structures with similar energies and similar mobilities (Supplementary Fig. S4). Overall, the solvent-exposed surface areas of key residues are also very similar (Supplementary Fig. S5). There is, however, a clear tendency for the 21–24 helix (which contains the critical Lys22 and Tyr23 residues) to adopt a different orientation with respect to the rest of the protein. This is illustrated in Fig. 4, which shows the distance between the Cα of Cys17 and Cα of Arg24, from which it is clear that the distance is consistently shorter when the disulfide dihedral angle is restrained to a positive value. The different conformations of the protein are illustrated further in Fig. 5, showing how the 21–24 helix adopts a different angle, putting the Lys22 and Arg24 sidechains in a different position. The only difference between the two molecular dynamics simulations is the restraint on the Cys17-Cys32 χ_3_ angle, but typically this restraint also led to changes in the Cys32 χ_1_ and χ_2_ angles (Fig. 5b), as expected from the analysis above.

## Discussion

Hydrostatic pressure increases the population of a conformation of ShK with a different structure from that observed in the previously-determined NMR structure. We can be confident that this is the same state populated at ambient pressure and responsible for binding to the potassium channel, for three reasons. First, because previous relaxation-based NMR studies at ambient pressure^15,20^ have identified an alternative state, which was hypothesised to be related to disulfide bond conformation. Second, because extensive research on protein folding has shown that proteins have evolved to have a very small number of low-energy minima^32^. This minimises the chances of aggregation, reduces the risk of misfolding, and ensures that the protein has the best probability of being in an active conformation. In most cases, it appears that the only stable local minima are conformations required for protein activity. It would thus be anomalous if ShK had two excited states, only one of which bound to the potassium channel. And third, because disulfide bond rotation is able to explain the observations made so far on ShK, as we now discuss.

The disulfide bond between two cystines is characterised by the χ_3_ dihedral angle, which is typically in a left- or right-handed conformation, with typical dihedral angles of ±80°. Extensive studies of bovine pancreatic trypsin inhibitor (BPTI) showed that one of the disulfides in BPTI, that between Cys14 and Cys38, undergoes slow exchange between two conformations that have different chiralities, associated with a different χ_1_ angle for Cys38, which switches from +60° in the major conformer to −60° in the minor conformer^33^. This conformational change can be described as a camshaft motion.

Camshaft motions of disulfides in proteins are not commonly observed. In BPTI, the alternative conformation is populated to only about 2%^33^. The exchange is slow enough to cause observable effects in the NMR spectrum, because BPTI is a very rigid and highly crosslinked protein. It seems likely that in most proteins, the exchange rate is too high, and the population of the alternative state too low, to lead to observable exchange^34^. ShK is highly crosslinked (three disulfides in 35 residues), which is likely to slow down conformational exchange. The population of the state with the alternative disulfide chirality is unusually high, raising the possibility of a functional role for this state.

In BPTI, the disulfide conformational change is coupled to a second fluctuation that leads to a flipping of the aromatic ring of Tyr35^33^. This is not surprising: inversion of the disulfide chirality produces a local conformational change that leads to further conformational change that propagates through the local structure and makes ring flips faster. Our molecular dynamics simulations show that an inverted chirality of the Cys17-Cys32 disulfide is accommodated into the structure with remarkably little structural change (Fig. 5b): the energies of the two conformations are indistinguishable, as are their dynamics, as evidenced by the fluctuations of the Tyr23 sidechain (see Supplementary Fig. S4).

There is, however, a small but significant conformational change resulting from the chirality change, of which the most obvious effect is that the short helix 21–24 is rotated by about 8°, in such a way that the sidechain of Lys22 points in a different direction. The sidechains of Arg24 and Tyr23 are also reoriented (Fig. 5c). This is a significant change, because previous studies^10,12^ have implicated the surface comprising His19, Lys22, Tyr23 and Arg24 as being critical for binding to the potassium channel, and in particular have identified Lys22 as being critical, with, for example, shortening of the Lys22 sidechain leading to a different conformation in the bound state^17^. Not surprisingly, removal of the Cys17-Cys32 disulfide produces a major loss in activity^35^.

The results presented here are therefore in agreement with previous structure-activity relationships. They are also consistent with the previous NMR dynamics studies^15,20^, which implicated the disulfides as being critical for the structural change. The relaxation dispersion study^15^ indicated chemical shift changes at a wide range of residues in ShK (18, 19, 21–25, 31 and 34) which do not form a contiguous surface. Our results explain this result, in that the disulfide inversion does cause structural rearrangements, but they are not confined to a single region. That study also calculated the ^15^N chemical shift changes fitted to the relaxation dispersion curves. We have compared those ^15^N shift changes to those calculated here, and the correlation is weak (*R* = 0.4) but present.

It is interesting to compare ShK with HsTX1, which has four disulfides, is considerably more rigid than ShK, and binds to Kv1.3 much more tightly than to Kv1.1^21^. It is possible that the selectively binding conformation is locked in to HsTX1, whereas it is only produced on binding to the channel in ShK.

Models of the complexes between potassium channels and their ligands are becoming increasingly sophisticated and predictive, thereby opening up the way to structure-based drug design^18^. However, modelling will only be successful if the starting structures are essentially correct. The results presented here, characterising the conformational difference between the free and bound structures, should help in improving the accuracy of the models, and thus their predictive power. High-pressure NMR is a useful way of characterising alternative and functionally relevant states^23,25^.

## Methods

^15^N-labeled ShK was produced as a thioredoxin fusion in *E. coli*, refolded and purified as previously described^36^. ^15^N and ^13^C double-labeled ShK was produced as a fusion with an N-terminal hexahistidine/maltose-binding protein (His_6_-MBP) tag by periplasmic expression in *E. coli* BL21(DE3)Gold. The sequence encoding the enterokinase (EK) cleavage site and ShK was subcloned using KpnI/NcoI restriction sites into a modified pET32a vector carrying the *malE* periplasmic signal sequence and His_6_-MBP cloned into the NdeI/KpnI sites. Cells transformed with the construct were grown in LB with shaking at 180 rpm, 37 °C to an optical density (OD_600_) of 0.6, before exchange into M9 medium containing 1 g L^−1^ NH_4_Cl and 3 g L^−1^ uniformly ^13^C-labeled glucose. The cultures were equilibrated for 1 h by shaking at 180 rpm at 25 °C, then induced using 0.4 mM IPTG and cultured overnight under the same conditions. The fusion protein was extracted by osmotic shock. Briefly, cell pellets were resuspended in 30%(v/v) sucrose, 100 mM Tris (pH 8.0), 2 mM EDTA, incubated at 4 °C for 20 min, then centrifuged at 8000 rpm for 20 min. The pellets were resuspended in 5 mM MgCl_2_ containing 1x cOmplete EDTA-free protease inhibitor cocktail (Roche), incubated at 4 °C for 30 min, then centrifuged at 16000 rpm for 20 min. The fusion protein was purified from the extract by nickel affinity chromatography on Chelating FastFlow Sepharose resin (GE Healthcare), and eluted using a step gradient of 100–300 mM imidazole. After dialysis into enterokinase cleavage buffer (20 mM Tris pH 8.0, 50 mM NaCl, 2 mM CaCl_2_), the His_6_-MBP tag was cleaved using enterokinase (New England Biolabs), and removed by cation exchange on a 7 × 25 mm HiTrap SP Separose FF column (GE Healthcare). Cation exchange fractions containing ShK were further purified by reverse phase high-performance liquid chromatography (RP-HPLC) as described previously^36^.

Peptide samples were dissolved in 10 mM sodium phosphate and 10 mM NaCl, pH 5.4, 10% D_2_O in H_2_O. High-pressure NMR studies were carried out on a Bruker Avance-III 600 MHz spectrometer fitted with a TXI cryoprobe, using a ceramic tube connected via pressure tubing to a high-pressure pump (Daedalus Innovations)^37^. Chemical shifts were measured using ^15^N HSQC, ^13^C HSQC and 2D HNCO spectra every 250 bar from 1 bar to 2500 bar. Proton chemical shifts were referenced to 3-trimethylsilyl-2,2,3,3-(^2^H_4_) propionate (TSP, Sigma Aldrich) at 0.0 ppm. Heteronuclear shifts were calculated relative to TSP using their gyromagnetic ratios^25^. Chemical shift assignments were taken from^38^.

Data analysis was carried out as described in^25^. Raw chemical shifts were processed using singular value decomposition (SVD) in MATLAB, in order to reduce experimental noise in the measured shifts. SVD components with rank larger than 4 were set to zero, and remaining values were used to calculate a ‘noise-free’ dataset. All chemical shift titrations were then rescaled to have the same maximum chemical shift change, and fitted to Eq. (1) using a Levenberg-Marquardt non-linear least-squares algorithm^39^. First, values for the global parameters Δ*G* and Δ*V* were obtained using a subset of resonances that displayed the largest pressure-dependent curvature, as estimated from χ^2^ values obtained from fitting to a simple quadratic equation (specifically, HN from I4, D5, T13, H19, R29; N from H19, Y23, C28, S30, C35; and CO preceding T13, M21, L25, C28 and T31). Subsequently, Δ*G* and Δ*V* values were fixed to these values, and residue-specific values for the chemical shift changes were obtained from fitting, after which the data were rescaled back to their original ranges. Backbone dihedral angles were obtained using TALOS-N^29^.

Molecular dynamics trajectories were calculated using the program CNS^40^, with TIP3 explicit water molecules. The calculations used an enhanced sampling scheme, in which structures were energy minimised, heated rapidly to 298 K (100 steps of 3 fs), and then run for 50,000 steps of 4 fs, followed by rapid quenching and energy minimisation. Thus, each iteration corresponded to 0.2 ns, and started from the end of the previous one. The trajectories started from the crystal structure (PDB code 4LFQ), which has a negative χ_3_ for the Cys17-Cys32 disulfide, restraining it to −80 ± 30°. For the alternative state, the starting coordinates were produced by restraining the disulfide dihedral to +80 ± 30°, followed by a short equilibration and energy minimisation. The dihedrals remained within their restrained angles throughout the trajectories.

## Supplementary information

Supplementary material

## Data Availability

The data supporting the findings of this study are available from the authors upon reasonable request.
