# Supplementary material for: Conformational exchange in the potassium channel blocker ShK
Source: Sci Rep. 2019 Dec 17;9:19307. doi: 10.1038/s41598-019-55806-3 (PMC6917819; doi:10.1038/s41598-019-55806-3)
Supplement: Supplementary file 1 — Supplementary material [file 41598_2019_55806_MOESM1_ESM.pdf]

Conformational exchange in the potassium channel blocker ShK

Naoto Iwakawa, Nicola J. Baxter, Dorothy C. C. Wai, Nicholas J. Fowler, Rodrigo A. V. Morales, Kenji Sugase, Raymond S. Norton and Mike P. Williamson

Supplementary Material

Five figures

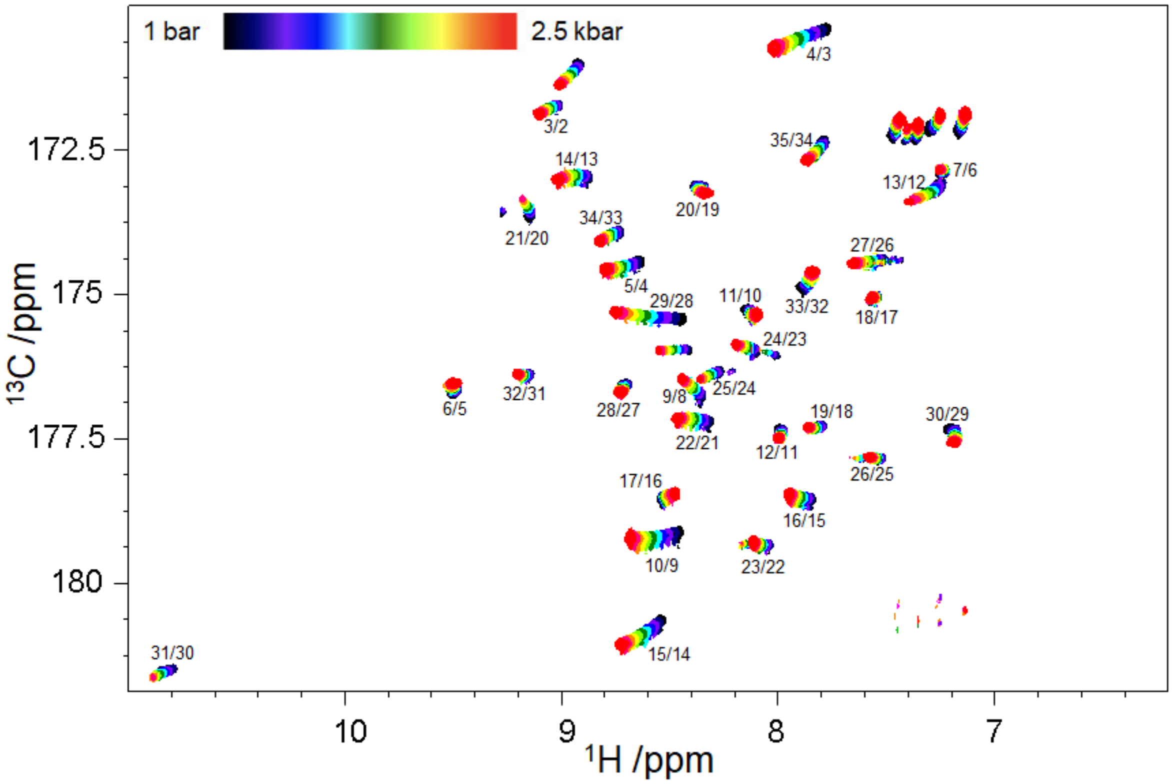

Fig S1a

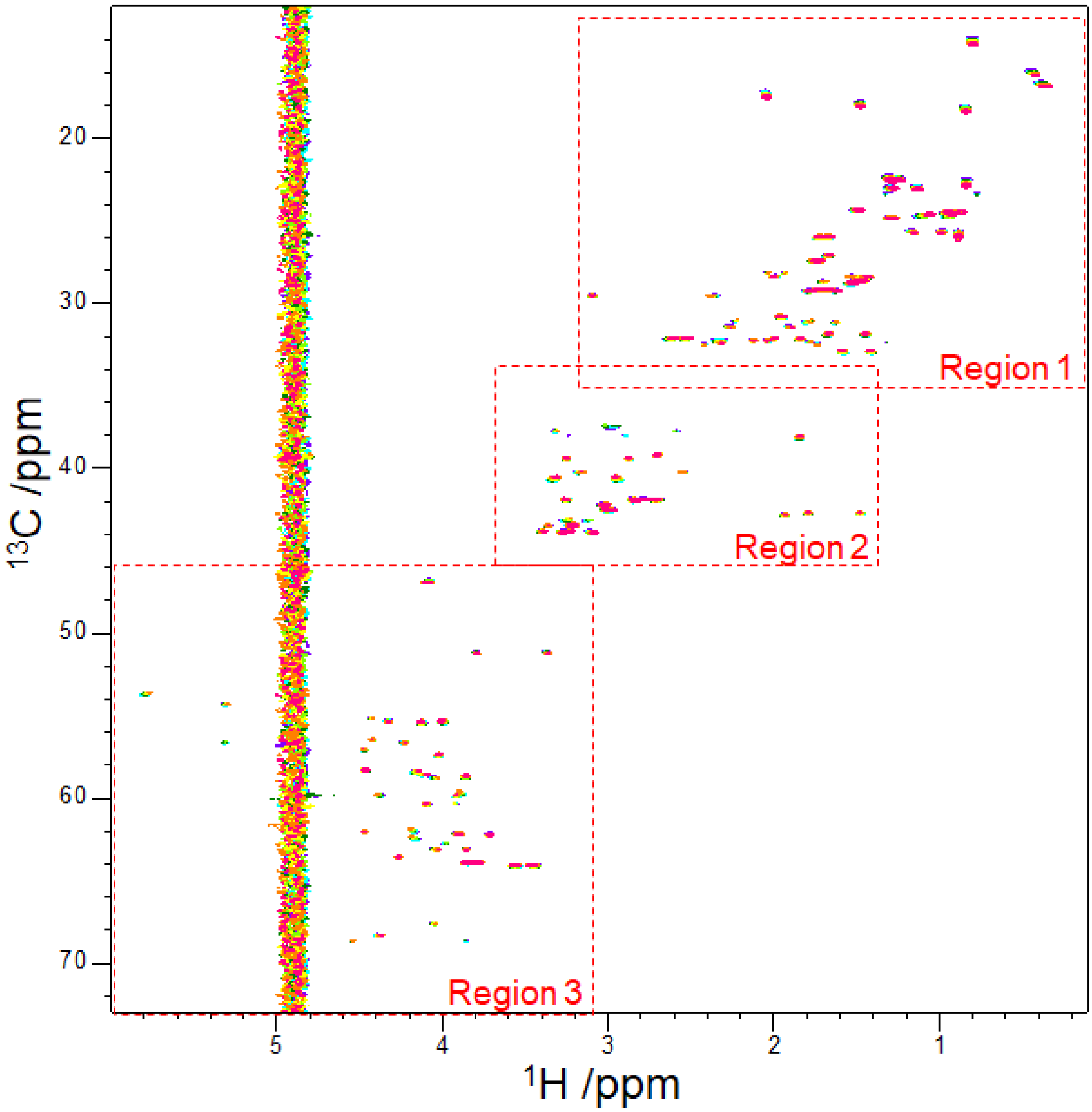

Fig S1b

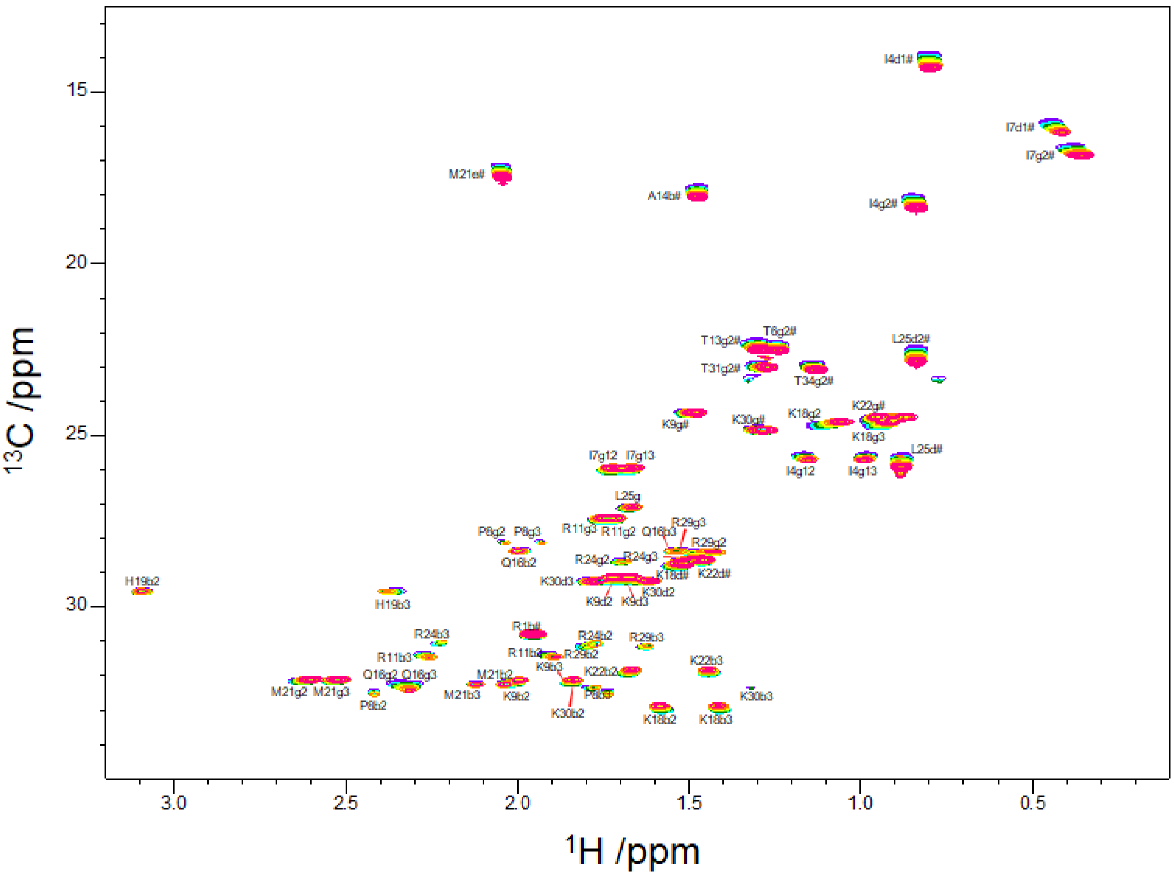

Fig S1c

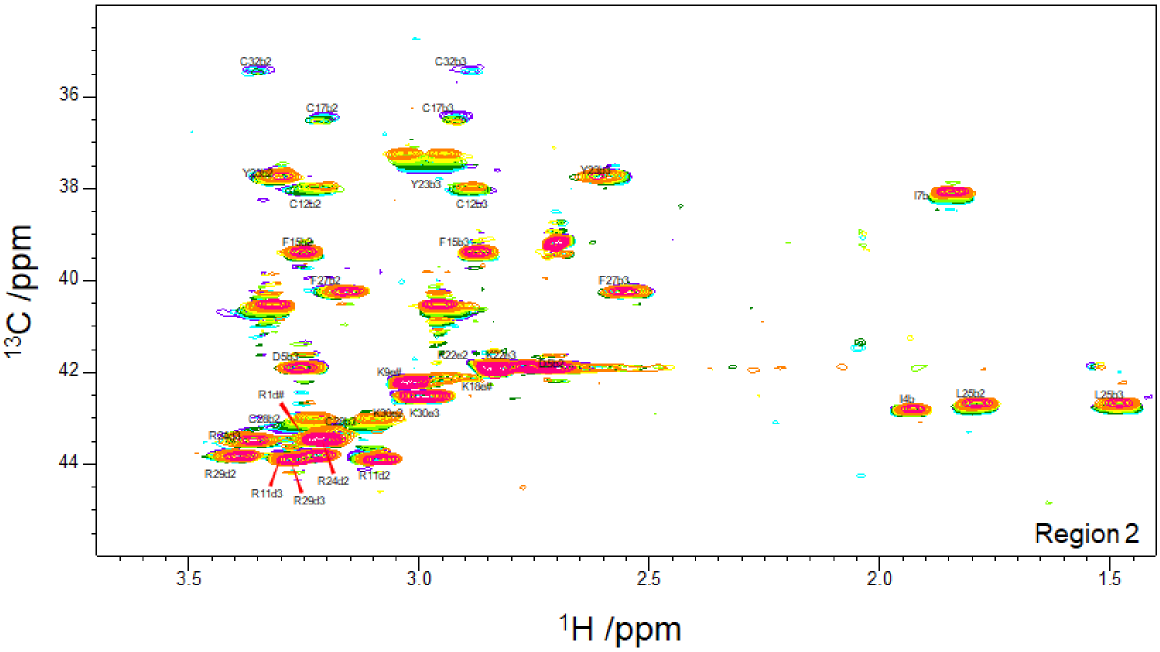

Fig S1d

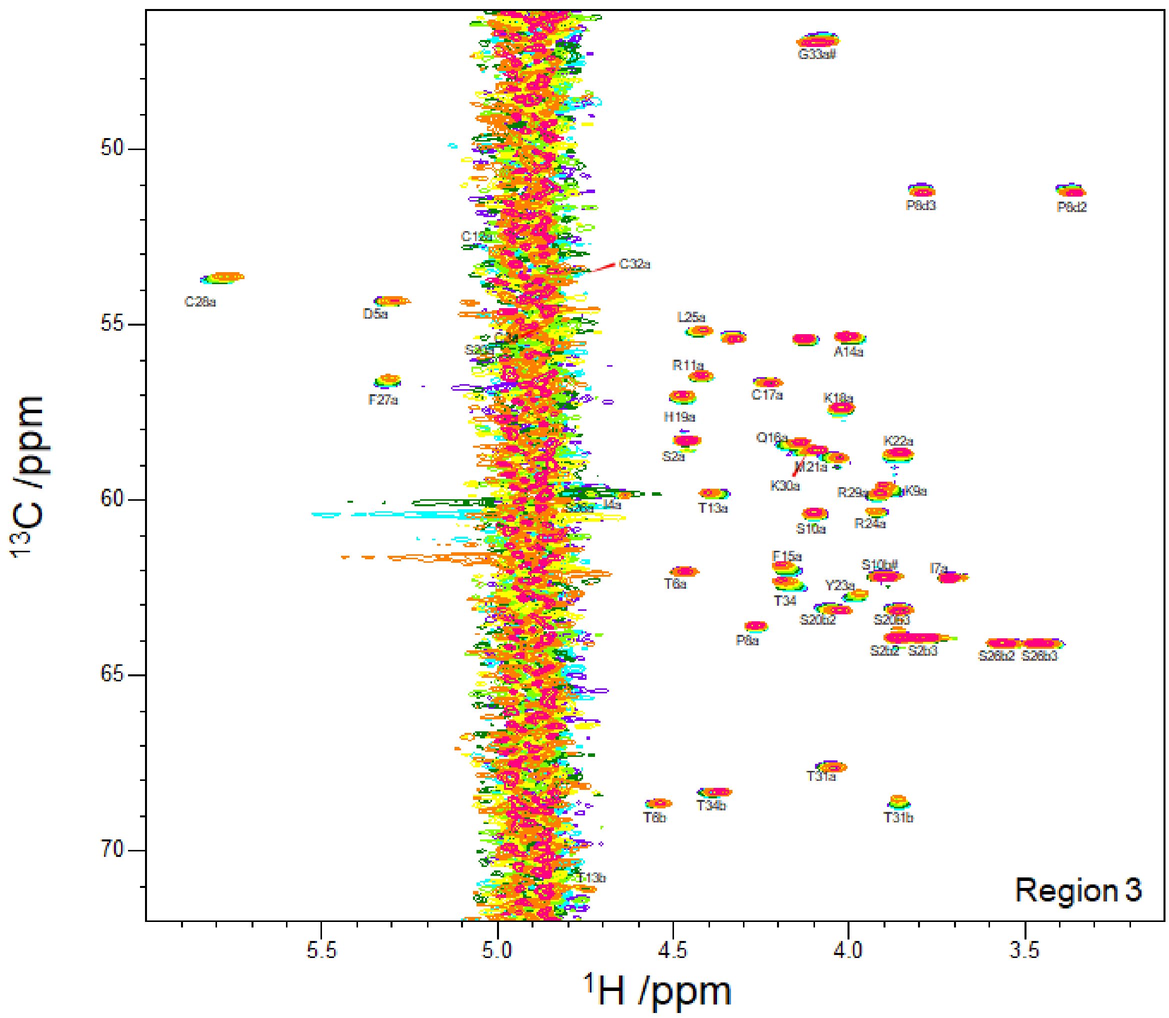

Fig S1e

Fig. S1. (**a**) Two-dimensional HNCO spectra of ShK acquired at pressures from 1 to 2500 bar. Peaks are labelled by the HN and CO residues. (**b**) ^{13}C HSQC spectra of ShK acquired at pressures from 1 to 2500 bar. The spectrum is subdivided into three regions, which are expanded in panels (**c**)-(**e**), with assignments.

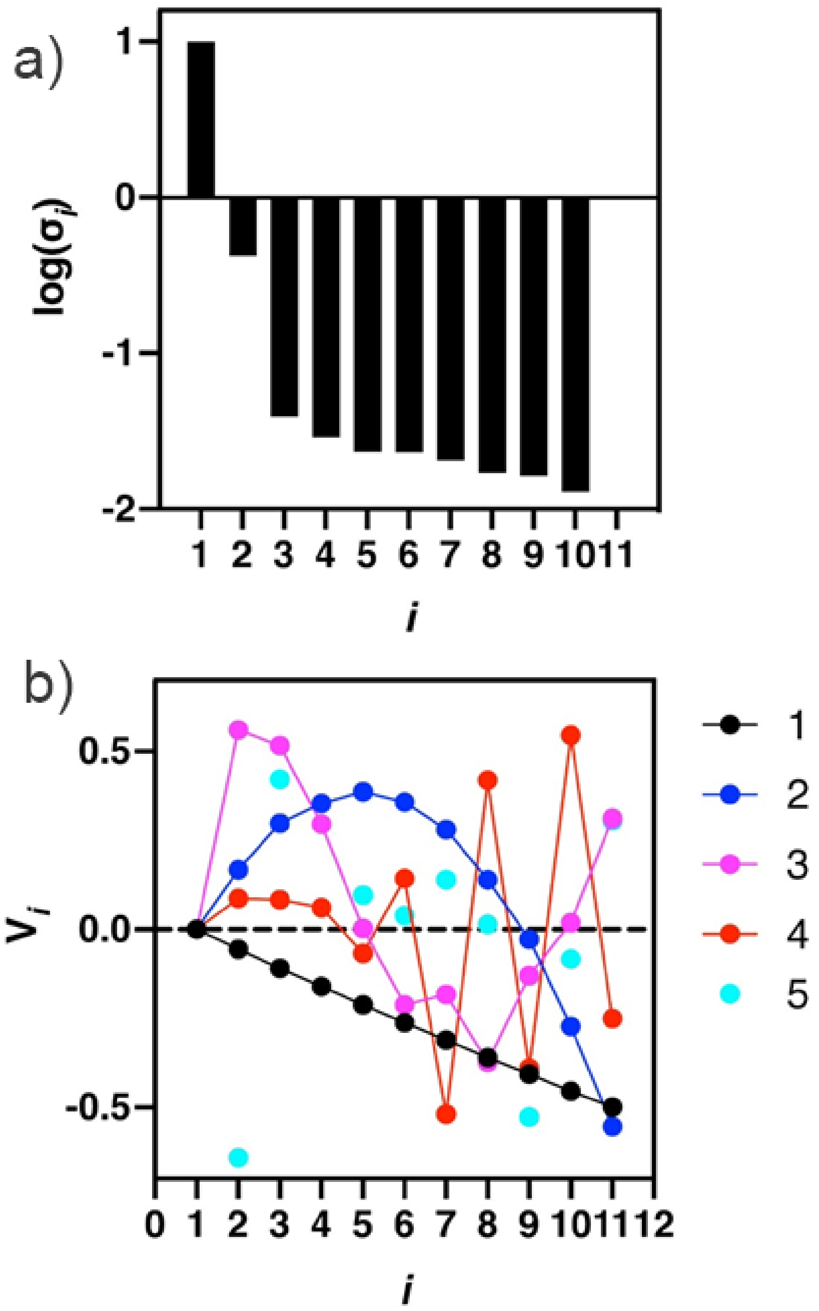

Fig. S2. (**a**) Singular values, obtained using singular value decomposition of the full set of ^{15}N and ^{1}H backbone amide shifts. The plot is of log (σ_i) *vs* *i*, where *i* is the index of the singular value, ranked in order from largest to smallest, and σ is the singular value. There are 11 singular values because there were 12 experimental pressures. It is clear that at least 3 singular values are required. For most signals, only three are required, because the curvature of shift with pressure can be fitted well to a quadratic. However, there is a small subset of resonances for which four values are clearly needed. (**b**) Plot of the first 5 of the column vectors of **V**, where **V** is the unitary matrix obtained from the SVD procedure **D** = **UWV**^{T}. Essential v_i should have smooth shape and high autocorrelation. It is clear that at least three singular values are needed.

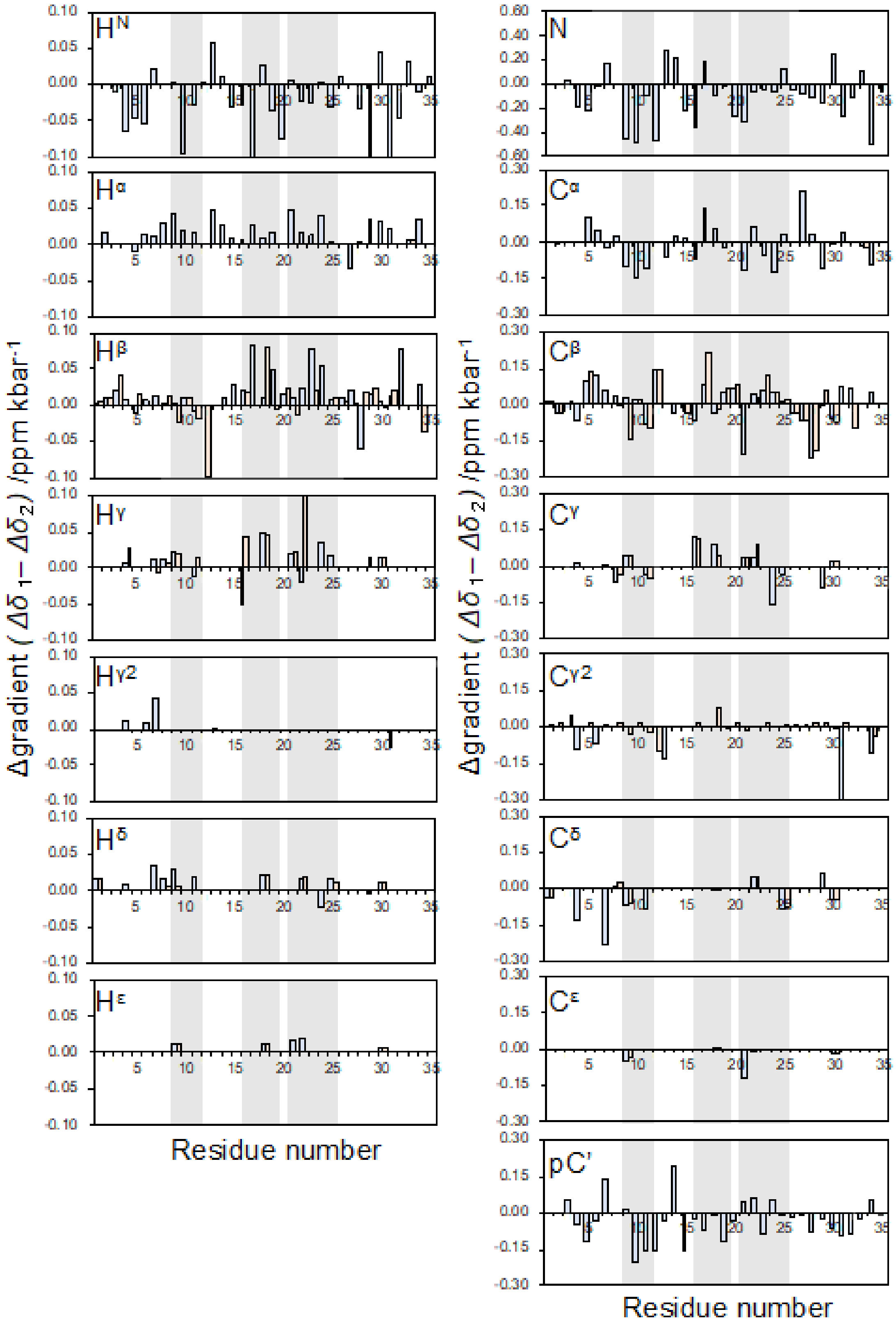

Fig. S3. Differences between the linear gradients $\Delta\delta_1$ and $\Delta\delta_2$ for ^{1}H, ^{15}N, ^{13}C′, ^{13}Cα and ^{13}Cβ shifts between the ground state and pressure-induced excited state. Regions of regular secondary structure are shaded. For carbons that have two protons attached, there are often two different shift values, given as cyan and orange bars.

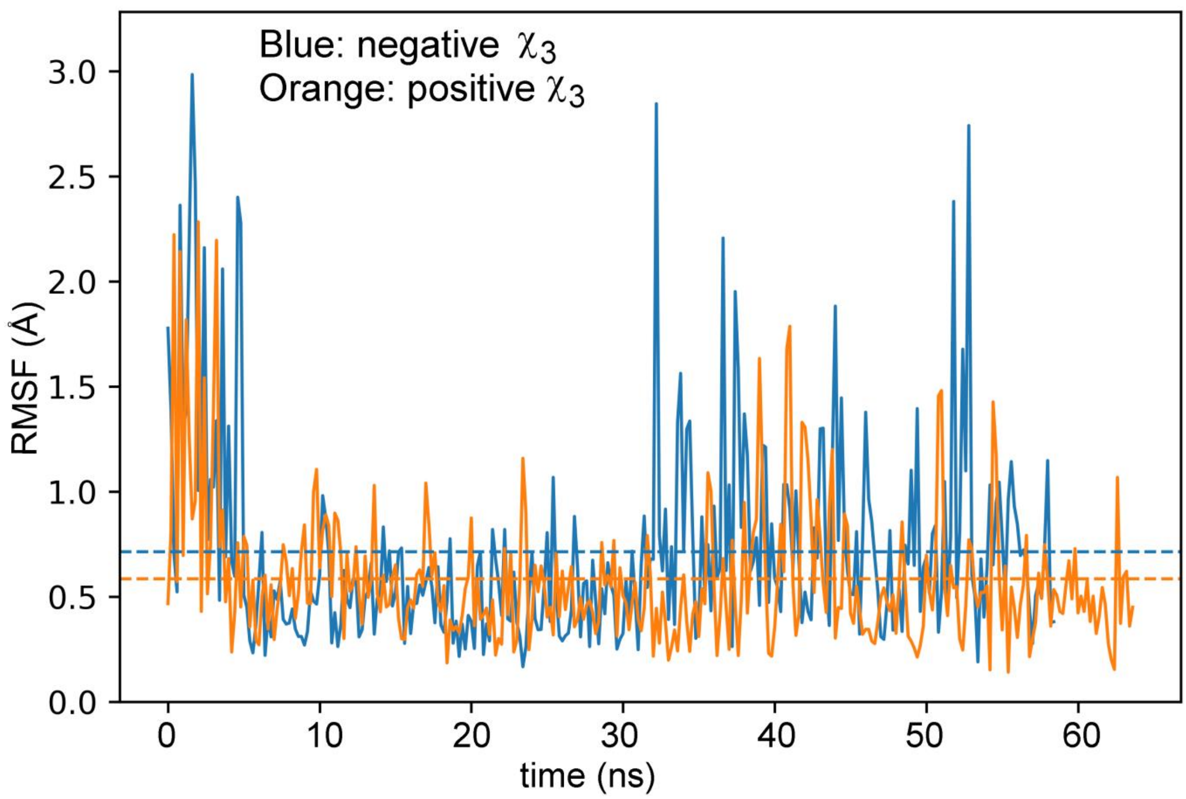

Fig. S4. Mobility of the Tyr23 sidechain, measured as the root-mean square fluctuation (Å) between one iteration and the next. The mean RMSF values are indicated by dashed lines, and have values 0.71 and 0.59 for negative and positive χ_3 respectively, where the dihedral in the ground state is negative, and in the alternative state is positive.

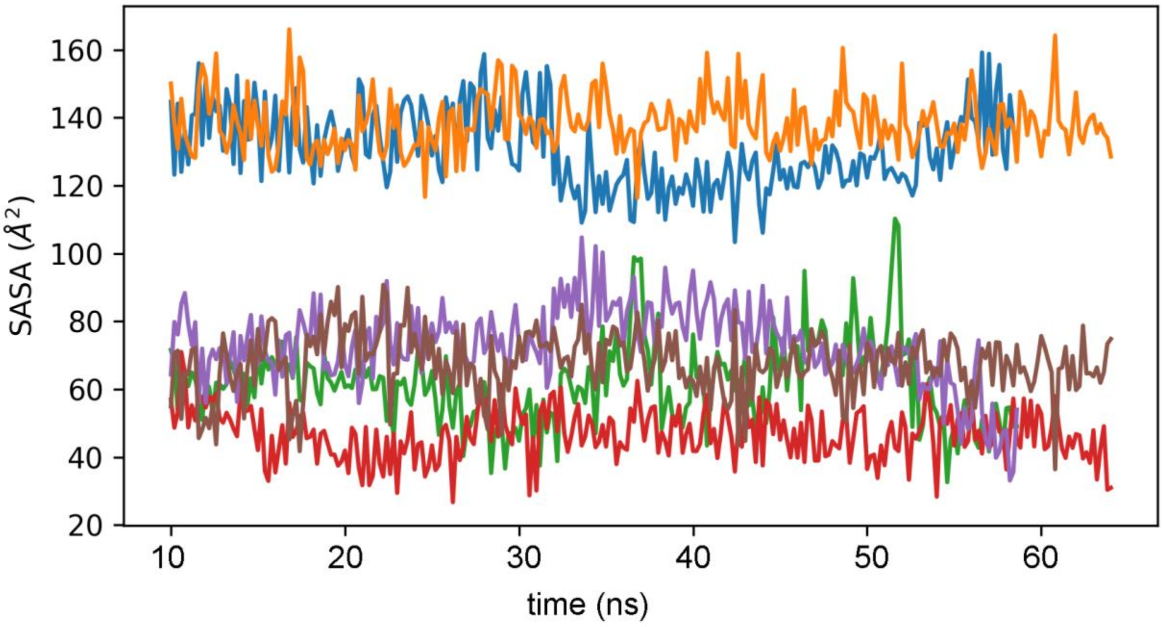

Fig. S5. Solvent-exposed surface area of the sidechains of three amino acid residues as a function of simulation time, for simulations in which the χ_3 disulfide dihedral angle was restrained to be negative (as in the crystal structure) or positive (as in the alternative conformation described here). The figure shows data for K22 (cyan: negative χ_3; orange, positive χ_3, mean values 131 and 138 Å^2 respectively), Y23 (green: negative χ_3; red, positive χ_3, mean values 62 and 47 Å^2 respectively), and R24 (purple: negative χ_3; brown, positive χ_3, mean values 73 and 68 Å^2 respectively).
